# Supplementary material for: Nutritional Composition and Microbial Communities of Two Non-alcoholic Traditional Fermented Beverages from Zambia: A Study of Mabisi and Munkoyo
Source: Nutrients. 2020 Jun 1;12(6):1628. doi: 10.3390/nu12061628 (PMC7352844; doi:10.3390/nu12061628)
Supplement: Supplementary file 1 [file nutrients-12-01628-s001.pdf]

Supplementary Table 1. Statistical analysis of differences in mean between measures for *Mabisi* and *Munkoyo* of nutritional parameters measured and pH (see Tables 1 and 2 in main text).

Parameter	Mabisi mean (SD)	Munkoyo mean (SD)	t-value	Degrees of freedom (df)	P-value
Moisture	85.34 % (3.61)	93.56 % (2.24)	-4.043	23	0.001
Dry Weight	14.67 % (1.81)	6.20 % (2.28)	-4.8	23	<0.001
Ash	0.67 % (0.047)	0.12 % (0.046)	21.765	23	<0.001
Fibre	0	0.62 % (0.124)			
Crude Protein	3.87 % (0.786)	0.36 % (0.175)	14.65	23	<0.001
Crude Fat	4.06 % (1.053)	0.84 % (0.262)	12.52	23	<0.001
Total Carbohydrate	6.07 % (6.57)	4.51 % (2.23)	2.884	23	0.008
Energy	76.28 % (31.901)	26.99 %(9.61)	8.123	23	<0.001
pH	4.12 % (0.17)	3.16 % (0.17)	14.392	23	<0.001
Vitamin B1	0.04 mg/100g (0.015)	0.036 mg/100g (0.019)	0.592	23	0.592
Vitamin B2	0.132 mg/100g (0.014)	0.038 mg/100g (0.01)	19.271	23	<0.001
Vitamin B3	0.329 mg/100g (0.136)	0.229 mg/100g (0.127)	1.906	23	0.069
Vitamin B6	0.022 mg/100g (0.005)	0.016 mg/100g (0.010)	1.865	23	0.075
Vitamin B12	0.394 μg/100g (0.077)	0			
Calcium	98.5 mg/100g (17.4)	3.654 mg/100g (2.106)	19.481	23	<0.001
Iron	0.188 mg/100g (0.154)	0.045 mg/100g (0.034)	3.076	23	0.005
Zinc	0.675 mg/100g (0.110)	0.274 mg/100g (0.062)	11.33	23	<0.001

Supplementary Table 2. Variation accounted for by the different nutritional parameters (in rotated space by Varimax with Kaiser Normalization)[a]. Principal Components 1 and 2 are shown with loadings of the nutritional parameters contribution to the respective components. Component 2 variation in the samples is explained by vitamins B1 and B6. Explain that loadings have a value between -1 and +1, zero being the average of all observations.

**Rotated Component Loadings[a]**		
	**Components**	
	**1**	**2**
Ash	0.957	
Vitamin B12	0.944	
Crude fat	0.944	
Vitamin B2	0.942	
pH	0.941	
Energy	0.936	
Ca	0.936	
Crude protein	0.921	
Zn	0.911	
Moisture	-0.813	
Total Carbohydrate	0.762	
Fe	0.708	
Vitamin B3	0.353	-0.283
Vitamin B6		0.875
Vitamin B1		0.865

Variable Principal Normalization. *a*. Rotation Method: Varimax with Kaiser Normalization.

Supplementary Table 3. Results of microbial community diversity analysis of traditional fermented foods *Mabisi* (MA) and *Munkoyo* (MU): alpha diversity measures (at highest sampling rarefaction of 15000 sequences per samples) for each sample based on Chao1 and Faith's Phylogenetic Diversity (PD).

Mabisi			*Munkoyo*		
**Sample ID**	**Chao1**	**Faith's Phylogenetic Diversity**	**Sample ID**	**Chao1**	**Faith's Phylogenetic Diversity**
MA1	309.2	2.9	MU1	148.4	2.4
MA2	470.4	2.8	MU2	142.4	2.2
MA3	206.2	2.3	MU3	103.8	2.0
MA4	339.1	3.4	MU4	155.5	2.0
MA5	471.1	3.2	MU5	98.9	2.0
MA6	262.4	2.4	MU6	197.1	3.4
MA7	357.0	2.6	MU7	85.8	2.1
MA8	185.9	1.7	MU8	140.2	2.4
MA9	341.3	2.6	MU9	88.0	3.9
MA10	289.8	2.5	MU10	223.1	2.9
MA11	283.5	2.3	MU11	95.5	2.3
MA12	311.5	2.6	MU12	117.1	2.2
			MU13	208.3	2.7
